# Mapping DNA Conformations Using Single-Molecule Conductance Measurements

**DOI:** 10.3390/biom13010129

**Published:** 2023-01-08

**Authors:** Mashari Alangari, Busra Demir, Caglanaz Akin Gultakti, Ersin Emre Oren, Joshua Hihath

**Affiliations:** 1 Department of Electrical Engineering, Engineering College, University of Ha’il, Ha’il 55476, Saudi Arabia; 2 Electrical and Computer Engineering Department, University of California Davis, Davis, CA 95616, USA; 3 Bionanodesign Laboratory, Department of Biomedical Engineering, TOBB University of Economics and Technology, Ankara 06560, Turkey; 4 Department of Materials Science and Nanotechnology Engineering, TOBB University of Economics and Technology, Ankara 06560, Turkey; 5 Biodesign Center for Bioelectronics, School of Electrical, Computer, and Energy Engineering, Arizona State University, Tempe, AZ 85287, USA

**Keywords:** single-molecule electronics, molecular electronics, single-molecule break junction, DNA, G-quadruplexes

## Abstract

DNA is an attractive material for a range of applications in nanoscience and nanotechnology, and it has recently been demonstrated that the electronic properties of DNA are uniquely sensitive to its sequence and structure, opening new opportunities for the development of electronic DNA biosensors. In this report, we examine the origin of multiple conductance peaks that can occur during single-molecule break-junction (SMBJ)-based conductance measurements on DNA. We demonstrate that these peaks originate from the presence of multiple DNA conformations within the solutions, in particular, double-stranded B-form DNA (dsDNA) and G-quadruplex structures. Using a combination of circular dichroism (CD) spectroscopy, computational approaches, sequence and environmental controls, and single-molecule conductance measurements, we disentangle the conductance information and demonstrate that specific conductance values come from specific conformations of the DNA and that the occurrence of these peaks can be controlled by controlling the local environment. In addition, we demonstrate that conductance measurements are uniquely sensitive to identifying these conformations in solutions and that multiple configurations can be detected in solutions over an extremely large concentration range, opening new possibilities for examining low-probability DNA conformations in solutions.

## 1. Introduction

DNA has attracted considerable interest in the nanoscience and nanotechnology communities in recent years. DNA’s unique structural, mechanical, and self-assembly properties yield new opportunities for nanometer-scale material placement and control, and its electronic properties have yielded interesting options for electronic devices and biosensing applications [1,2,3]. Recently, the single-molecule break-junction (SMBJ) technique has arisen as a promising tool for probing the single-molecule conductance value for various DNA and RNA sequences [4], their conformations [5], environmental effects [6], and base mismatches [7]. The detection of biologically relevant RNA duplex structures at extremely low concentrations is one of the great advantages that SMBJ offers [8]. However, the development of real applications that utilize DNA as a platform for electronic systems, whether as a component of lithography or as independent devices, requires an in-depth understanding of the interplay between the electrical properties and structural conformations of the DNA systems. While DNA is typically regarded as a straight-forward right-handed double helical structure, the conformation space is far more complex. There are a number of minima in the energy space that give rise to a diverse array of stable conformations for DNA (B-form, A-form, Z-form, G-quadraplex, i-motif, etc.) depending on the sequences and the local environment [9,10]. However, even within these categories, a significant number of stable or meta-stable conformations that are significantly different from the textbook conformations can occur. From an electronic device point-of-view, the result of such a complex space-state for DNA is electronic properties that vary widely for a given sequence depending on the conformation and local environment.

In this work, we disentangle the electronic properties of a set of DNA sequences that can form different DNA structures in solution and demonstrate that the SMBJ approach can distinguish between these double-stranded B-DNA (dsDNA) and G-quadraplexes in a mixed solution with better fidelity than traditional approaches such as circular dichroism (CD) spectroscopy. In addition, we performed molecular dynamics (MD) and electronic structure calculations for both structures to examine their stability and HOMO delocalization. Moreover, we demonstrate that by controlling the local environment, we can controllably modify the structures to obtain only a single conductance value during the break-junction measurements and that we can detect both conformations in the solution over a wide ratio of concentrations.

## 2. Experimental Procedure

### 2.1. DNA Sample Preparation

The oligonucleotides investigated were purchased from a variety of vendors; 5′-(CG_3_)_3_-3′-C_3_H_6_-SH and 5′-(C_3_G_3_)_2_-3′-C_6_H_12_-SH were purchased from Biosynthesis (Lewisville, TX, USA). Non-thiolated oligonucleotides were purchased from Alpha DNA and IDT. Molecules 5′-G_3_-T_3_-G_3_-3′-C_3_H_6_-SH and 5′-CCGC GAG CGG-3′-C_3_H_6_-SH and their non-thiolated versions were purchased from IDT (USA). In each case, the molecules were purified using high-performance liquid chromatography (HPLC). All DNA strands were stored at −80 °C. Prior to measurements, tris(2-carboxy-ethyl)phosphane (TCEP, 12.5 mM) was used to reduce the disulfide bonds on the thiolated strands for 3 h at room temperature. Then, the excess and unreacted TCEP was removed using 7k molecular-weight cutoff desalting spin columns (ThermoFisher scientific Zeba # 89882, Waltham, MA, USA). All experiments were conducted in a 100 mM sodium phosphate-buffered solution (PBS) or a 100 mM PBS with 100 mM of potassium chloride (KCl) added. The 100 mM PBS was prepared by adding Na_2_HPO_4_ and NaH_2_PO_4_ (Sigma-Aldrich, St. Louis, MO, USA) in a ratio of 8.1/1.9 to obtain a 7.4 pH solution. The KCl-containing PBS solution was prepared by adding KCl to 100 mM PBS to obtain a KCl concentration of 100 mM. The final solution had a pH of 7.4. All solutions were prepared using Milli-Q water (18 MΩ). Hybridization was obtained by heating the mixture to 90 °C and then cooling it to 23 °C at a rate of 1 °C/minute. Then, the mixture was stored at −20 °C.

### 2.2. CD Experiment Set-Up

CD measurements were conducted using an Olis RSM 1000 circular dichroism spectrometer (Olis, Inc., Bogart, GA, USA) with a cylindrical cell (170 µL) and 0.1 mm path length. A baseline spectrum of the buffer only was collected before adding the DNA. Non-thiolated hybrids were prepared using the above-mentioned annealing protocol. A 170 µL of a 25 µM DNA solution was added to the cylindrical cell.

### 2.3. SMBJ Measurements and Data Analysis

We measured single-molecule conductance using the SMBJ approach. The gold substrates were prepared by the thermal evaporation of 130 nm gold on freshly cleaved mica surfaces. Prior to each experiment, the substrate was briefly flame-annealed using a butane torch. The tip electrode was prepared by cutting a 0.25 mm gold wire (Alfa- Aesar, 99.998% purity, Haverhill, MA, USA) and then coating it with Apiezon wax to reduce the leakage current to ~pA levels. Piranha, a strong, corrosive and oxidizing solution, was used to clean a Teflon cell that held the solvent for the measurements. All experiments were performed using a Molecular Imaging Pico-STM head connected to a modified Digital Instruments Nanoscope IIIa controller (Digital Instruments Inc., Santa Barbara, CA, USA) at room temperature. A Labview program (National Instruments) was used to control the tip electrode using a PCIe-6363 DAQ card (National Instruments).

The conductance measurements started by moving the tip into the substrate until the 10 nA/V preamplifiers saturated. Then, the tip was retracted at a rate of ~ 80 nm/s while the current traces were recorded at a fixed bias until the current reached the lower limit of the preamplifier (~10 pA). This process was repeated to collect ~5000 traces. Prior to DNA conductance measurements, control experiments were taken in a buffer solution before adding DNA molecules into the Teflon cell, which allowed us to verify that no contamination was present. Unless otherwise specified, for conductance measurements, a ~ 1µM final DNA concentration was achieved by adding a small volume of DNA molecules into the cell. Most of the single traces show exponential decay without steps, which suggests no molecular bridge between the gold electrodes. However, some of the traces between 10 and 15% show steps and are added to the histogram using an automated Labview program (see Table S1 for the percentages of the selected traces in titration measurements).

During the data screening process, an automated LabView program was used to select traces to add to the composite conductance histogram. The first criterion allowed the exclusion of exponential decay traces by linearly fitting each trace on a semi-logarithmic plot, and traces with a fitting residual below a preset value were rejected. Then, a threshold peak amplitude in individual histograms was specified to determine which traces were added to the histogram. Traces with a step-like feature show a sharp spike in the individual histograms. All the selected curves were added together to obtain a semi-logarithmic conductance histogram, which revealed the most probable conductance of that molecular junction.

### 2.4. Molecular Dynamics and Electronic Structure Calculations

Double-stranded DNA molecules are generated using an AMBER Nucleic Acid Builder. The G-quadruplex structure is generated by modifying the X-ray structure PDB ID: 5hix. Both structures are neutralized with Na^+^ counterions. The four K+ ions in between the G-quartets were placed in the same positions as in the template for the G-quadruplex structure. Both structures were placed into an octahedral water box which had a 15 Å cutoff from the DNA molecules. First, water molecules and counterions were subjected to 500 steps of energy minimization, while DNA molecules were restrained with 50 kcal/mol force. Then, 5000 steps of energy minimization were applied to the entire system without any restraint on any molecule. Then, the system was heated to 300 K in an NVT ensemble within 100 ps while a 50 kcal/mol restraint force was applied to the DNA molecules. Next, the system equilibrated for 100 ps while a 0.5 kcal/mol restraint force was applied only to the DNA molecules. Finally, the entire system was simulated in an NPT ensemble for 100 ns without any restraining force applied via the AMBER 14 [11] pmemd CUDA module. The force fields bsc1 [12] and TIP3P [13] were employed to describe the DNA, water molecules, and counterions in each simulation. The particle Mesh Ewald [14] algorithm was used for long-range electrostatic interactions, and a cut-off value of 10 Å was applied to the van der Waals interactions. The simulations were performed and recorded for every 2 fs, and the SHAKE algorithm [15] was implemented for all bonds with the hydrogen atoms.

We employed the VMD [16] software’s (Theoretical Biophysics Group, University of Illinois, Urbana, IL, USA, version 1.9.4a57) root mean square deviation (RMSD)-based clustering approach and categorized all conformations to find the most stable one in the simulation time period. Next, the representative structures were subjected to energy minimization before electronic structure calculations. Water molecules and counterions were removed from the minimized structures, and the total charge of each system was set to −22. Then, the density functional theory calculations were carried out using the Gaussian 16 [17] software package (Gaussian, Inc., Wallingford, CT, USA, version 16) with the B3LYP exchange-correlation function and 6–31G(d,p) basis set together with the polarizable continuum model. Molecular orbitals were plotted using the Avogadro [18] Software program (an open-source molecular builder and visualization tool. version 1.2.0, http://avogadro.cc/, accessed on 28 November 2022).

## 3. Results and Discussion

**Figure 1:**
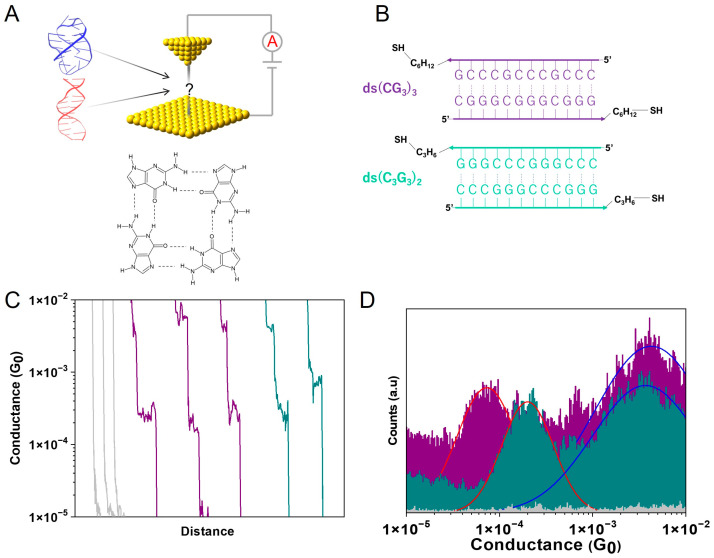
(**A**) Schematic of the SMBJ setup, a planar G-tetrad, and G-quadruplex (blue) and B-form dsDNA (red) structures. (**B**) DNA sequences used in this experiment in their double-stranded configuration, ds(CG_3_)_3_ (purple) and ds(C_3_G_3_)_2_ (dark cyan). (**C**) Multiple single-molecule conductance vs. distance traces at room temperature. The gray traces demonstrate when no molecules bind to the electrodes, and the purple and green are for (CG_3_)_3_ and (C_3_G_3_)_2_ when binding to electrodes, respectively. All traces were offset horizontally for clarity. (**D**) Conductance histograms for (CG_3_)_3_ (purple), (C_3_G_3_)_2_ (dark cyan), which show two conductance peaks each, and the control experiment for blank buffer. The blue and red lines are gaussian fittings to the peaks (see Supplementary File).

To begin, we performed SMBJ-based conductance measurements on two 12-base pair sequences, (CG_3_)_3_ + complement and (C_3_G_3_)_2_ (note both sequences have a thiol linker, as shown in Figure 1B). SMBJ experiments proceeded by applying a bias between two electrodes and measuring the current between them while moving the electrodes into and out of contact in the presence of molecules that were capable of binding between the two electrodes [19]. As the two electrodes were separated, there was some probability that a molecule would bind between the two electrodes; when this occurred, plateaus were observed in the current vs. distance trace (Figure 1C), but when no molecules were bound, the trace appeared as an exponential decay. By repeating these electrode separation measurements thousands of times, it was possible to perform a statistical analysis of the step position to obtain the most-probable conductance value from a histogram, as shown in Figure 1D. In the cases of (CG_3_)_3_ and (C_3_G_3_)_2,_ two distinct peaks were clearly visible in the conductance histograms indicating that there were two highly probable conductance values for these molecules. Interestingly, the high-conductance peak was similar in both cases ~3.55 ± 0.42 × 10^−3^ *G*_0_ (where *G*_0_ is the conductance quantum equal to 77.48 µS), while the lower conductance peak was significantly different in the two cases, 1.63 ± 0.4 × 10^−4^ *G*_0_ for (CG_3_)_3_ and 2.31 ± 0.1 × 10^−4^ *G*_0_ for (C_3_G_3_)_2_; the quoted error range for each measurement is given by the standard deviation of the peak position from three independent measurements.

We have previously shown that different conductance peaks can appear for DNA structures because of changes in the conformation (e.g., A-form vs. B-form) [20]. In this case, because of the repeating G-triplets within each of the oligonucleotide sequences, we hypothesized that high conductance peaks would originate from the formation of G-quadraplexes in the solution, which could readily form in these guanine-rich sequences [21,22] and that the other would come from double-stranded (ds)DNA. With this hypothesis, we will refer to double-stranded versions of each of the two sequences as ds(CG_3_)_3_ and ds(C_3_G_3_)_2_, G-quadruplex versions as (CG_3_)_3_-G-quad, and (C_3_G_3_)_2_-G-quad, and solutions containing both configurations as simply (CG_3_)_3_ and (C_3_G_3_)_2_.

G-quadruplexes are formed by stacking multiple guanine tetrads, which are the planar association of four guanines via Hoogsteen hydrogen bonding (Figure 1A) [23]. In general, depending on the sequence, loop length, and the ions present, the strand directions in G-quadruplexes can vary, and parallel, antiparallel, or hybrid (3+1) topologies are possible [24,25]. These structures can be formed from either a single strand (intramolecular) or multi-strands (intermolecular), and the fact that we could see these two conductance values in two significantly different sequences suggests this may be a general effect in SMBJ measurements. Identifying, understanding, and tracking G-quadraplexes has become increasingly important in recent years since it has been shown that they are responsible for regulating multiple biological processes in vivo [26,27], and they are now being studied as drug targets for multiple diseases, including cancers and HIV [28,29]. However, despite this interest, it is still often difficult to identify and quantify the number of G-quadraplexes present in a DNA mixture [30,31,32].

**Figure 2:**
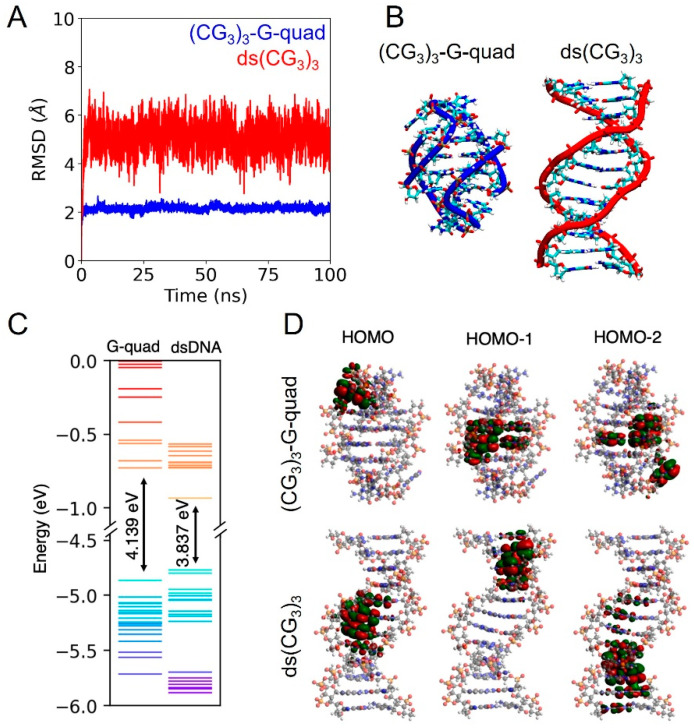
(**A**) Root mean square deviation (RMSD) for ds(CG_3_)_3_ (red line) and (CG_3_)_3_-G-quad (blue line). (**B**) (CG_3_)_3_-G-quad and ds(CG_3_)_3_ structures that are representative of the ones that were the most stable throughout the course of the MD simulation. (**C**) One-dimensional energy level plot for (CG_3_)_3_-G-quad and ds(CG_3_)_3_. (**D**) Three-dimensional iso-surface plot of the HOMO, HOMO-1, and HOMO-2 orbitals for (CG_3_)_3_-G-quad and ds(CG_3_)_3_ structures.

To first test this hypothesis, we began by performing a combination of molecular dynamics (MD) and density functional theory (DFT) calculations, as shown in Figure 2. For the MD calculations, we ran 100 ns simulations of both the dsDNA and G-quadraplex forms of (CG_3_)_3_. As can be seen in Figure 2A, both of these forms are stable in the solution, with the G-quadruplex configuration having a much lower mean-square deviation, indicating a very stable configuration. In addition, from the DFT calculations we can see that even though the energy gap between the highest occupied molecular orbital (HOMO) and lowest unoccupied molecular orbital (LUMO) is slightly larger in the case of the G-quadruplex structure (Figure 2C), the energy levels are more delocalized between the two contact points in the quadruplex case, suggesting a higher overall conductance (Figure 2D).

**Figure 3:**
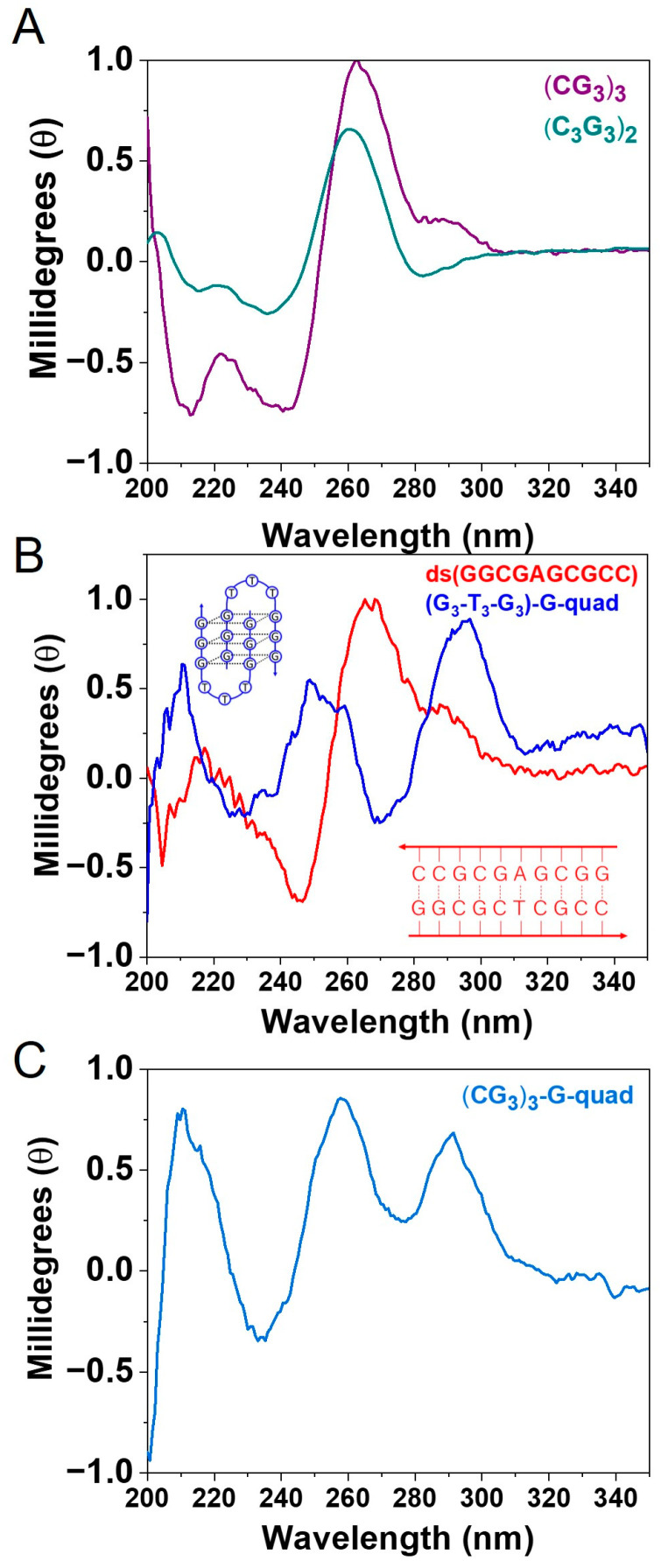
Circular dichroism spectra measured in 100 mM PBS for (**A**) (CG_3_)_3_ (purple) and (C_3_G_3_)_2_ (dark cyan). (**B**) CD spectrum for ds(GGCGAGCGCC), red line, with a B-form helical structure, which is distinguished by a positive band between 260 and 280 nm and a negative band near 245 nm. The blue line is the CD spectrum for the antiparallel configuration of (G_3_-T_3_-G_3_)-G-quad with a positive band at 290 nm; inset: schematic representations of ds(GGCGAGCGCC) (red) and (G_3_-T_3_-G_3_)-G-quad (blue). (**C**) The light blue line depicts (CG_3_)_2_-G-quad hybridized in solution without the complementary strand, resulting in a G-quad structure with a positive peak at 290 nm.

Having demonstrated that both of these structures are stable for this sequence, we now attempted to experimentally verify our hypothesis by performing CD spectroscopy on the various sequences. CD is one of the primary methods for understanding the global features of the DNA structure and the topology of G-quadruplexes. [33,34]. CD spectra provide insights into DNA conformations by measuring the absorbance difference between right and left circularly polarized light [35]. In the case of (CG_3_)_3_ and (C_3_G_3_)_2,_ the CD response shown in Figure 3A is ambiguous, as it does not follow the trends expected for a textbook, A, B, or Z-form duplex, or a G-quadraplex (see Figure 3B for examples of B-form and G-quadraplex) [36,37]. This ambiguity likely stems from having multiple configurations contributing to the measured ellipticity in the spectrum [38].

Therefore, as an alternative test of the hypothesis, we directly measured specific, well-controlled molecular configurations using both SMBJ conductance measurements and CD spectroscopy. As shown in Figure 3, we used the sequence CCGC GAG CGG-C_3_H_6_-SH + complement to create a B-form dsDNA duplex, which will be referred to as ds(GGCGAGCGCC), and G_3_-T_3_-G_3_-C_3_H_6_-SH to form a G-quadraplex which will be referred to as (G_3_-T_3_-G_3_)-G-quad. The CD spectra for each of these cases match well with the expected spectra for B-form and G-quadraplex DNA structures (Figure 3B, red and blue, respectively) [39,40]. The B-form is characterized by a positive band in the 260–280 nm range and a negative band around 245 nm [35]. The blue spectrum in Figure 3B suggests that the sequence (G_3_-T_3_-G_3_)-G-quad adopts the antiparallel conformation G-quadruplex structure. The positive band at a 295 nm wavelength is a signature for antiparallel conformation [33,41].

**Figure 4:**
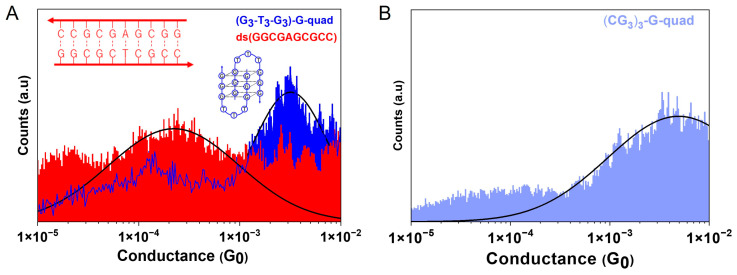
Break junction measurements on (**A**) 5′-G_3_-T_3_-G_3_-G-quad (blue) and dsCCGCGAGCGG (red); inset: schematic representations of dsDNA and G-quadruplex. (**B**) Conductance histogram for the (CG_3_)_3_-G-quad structure, which is obtained by hybridizing the sequence (CG_3_)_3_ without the complementary strand present.

The conductance histograms for these sequences are shown in Figure 4. The conductance value for each molecule was obtained by fitting individual conductance histograms from at least three independent measurements with a Gaussian distribution. The fit yielded conductance values of 3.03 ± 0.42 × 10^−4^ *G*_0_ and 3.77 ± 0.16 × 10^−3^ *G*_0_ for the ds(GGC GAG CGCC) and (G_3_-T_3_-G_3_)-G-quad sequences, respectively. Given that the conductance of this 10-base-pair GC-rich sequence is within the range of the lower conductance peaks observed for (CG_3_)_3_ and (C_3_G_3_)_2_ and that the conductance of the 3-base G-quadruplex is within the range of the higher conductance value for these sequences, we thereby ascribe the lower conductance peak to the double-stranded duplex conformations of each sequence, and the higher conductance to the G-quadruplex structure. This matched well with our expectations since the G-quadruplex structures are significantly shorter than the corresponding DNA duplexes.

To provide further evidence for this peak assignment, we also studied the CD spectrum and conductance values for the (CG_3_)_3_-G-quad. If this sequence is hybridized in a solution without the complement present, it would be expected to exclusively create G-quadruplex structures. Additionally, from the CD spectrum in Figure 3C, it is clear that this sequence adopts an antiparallel G-quadruplex topology, which is identified by the presence of the 295 nm, 210 nm, and 260 nm positive peaks [33,34]. This spectrum matches well with the (G_3_-T_3_-G_3_)-G-quad sequence, corroborating the assignment of this structure as a G-quadruplex. In addition, the conductance for this sequence (Figure 4B) was found to be 3.5 ± 0.2 × 10^−3^ *G*_0_, which is within the range of the higher conductance value seen in Figure 1D when the complement is present, thus providing clear evidence that the high conductance peaks seen in Figure 1D are due to G-quadruplex structures.

**Figure 5:**
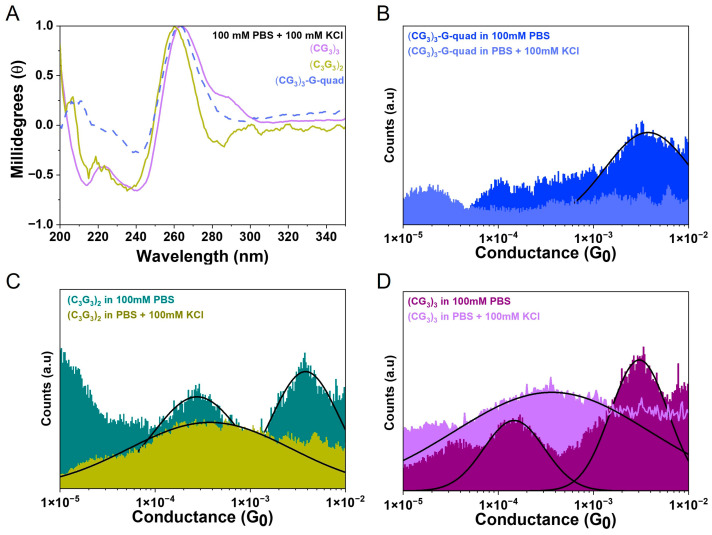
CD and conductance histograms for DNA hybrids (**A**) CD spectra in 100 mM PBS with 100 mM KCl for (CG_3_)_3_-G-quad (dashed line), (CG_3_)_3_ (light magenta) and (C_3_G_3_)_2_ (dark yellow). (**B**) conductance histograms for (CG_3_)_3_-G-quad in 100 mM PBS (blue) and 100 mM PBS + 100 mM KCl (light blue). (**C**) Conductance histograms for (C_3_G_3_)_2_ in 100 mM PBS (dark cyan) and 100 mM PBS + 100 mM KCl (dark yellow). (**D**) Conductance histograms for (CG_3_)_3_ in 100 mM PBS (purple) and 100 mM PBS + 100 mM KCl (light purple). All peaks are fit with a gaussian distribution in log scale.

For the (CG_3_)_3_ sequence, we were able to unambiguously identify the individual peaks because the system was not self-complementary, thus allowing us to test the G-quadruplex structure independently. However, for the (C_3_G_3_)_2_ sequence, it is not possible to directly obtain (C_3_G_3_)_2_-G-quad because it is self-complementary. Therefore, to independently verify the peak assignments, we further examined the environmental control over the molecular configurations. It has previously been demonstrated that potassium ions are able to destabilize the antiparallel G-quadruplex topology in favor of the parallel topology, which is believed to be due to the size of the potassium ions [42,43]. Here, we added 100 mM KCl to our sodium phosphate-buffered solution (PBS) to attempt to control the observed conformations in the break junction system. The addition of the K^+^ ions significantly changed the CD spectrum for (CG_3_)_3_-G-quad, which now resembled that of a parallel conformation quadruplex (Figure 5A (dashed line)). Slight changes were apparent in the CD spectra for both the (C_3_G_3_)_2_ and (CG_3_)_3_ solutions at the 295 nm wavelength (Figure 5A), but these changes were less dramatic than for the (CG_3_)_3_-G-quad since these solutions were still mixtures with both duplexes and quadruplexes present. Although CD spectroscopy is one of the primary methods for understanding the structure of DNA, it is clear from this analysis that the CD spectra are often not conclusive because of the difficulty in the convolution of the signal.

Alternatively, the SMBJ-based conductance features change dramatically with the addition of KCl to the solution. In the parallel topology, the thiols are both on one end of the quadruplex and, as such, may bind to a single electrode, making the probability of junction formation much lower. Additionally, if a junction is formed, in this configuration, the transport would have to be in the transverse direction, which is significantly different from what occurs in the antiparallel configuration. The SMBJ measurements on the (CG_3_)_3_-G-quad in 100 mM KCl show no significant plateaus in the current traces and yield no peak in the conductance histogram (Figure 5B, light blue). The conductance histograms obtained for (CG_3_)_3_ and (C_3_G_3_)_2_ in a 100 mM KCl solution now show only a single conductance peak (Figure 5C,D). The conductance values are 3.13 ± 0.8 × 10^−4^ *G*_0_ and 3.36 ± 0.3 × 10^−4^ *G*_0_ and relate to the expected values for ds(CG_3_)_3_ and ds(C_3_G_3_)_2_, respectively. These values are similar to but slightly different from those obtained in 100 mM PBS, which is probably due to small differences in the B-form conformation in the presence of different ions [44,45]. These experiments demonstrate that not only is the origin of the multiple peaks due to the different configurations of the molecule but also show that the conformations can be controlled and tracked with SMBJ-based conductance measurements.

**Figure 6:**
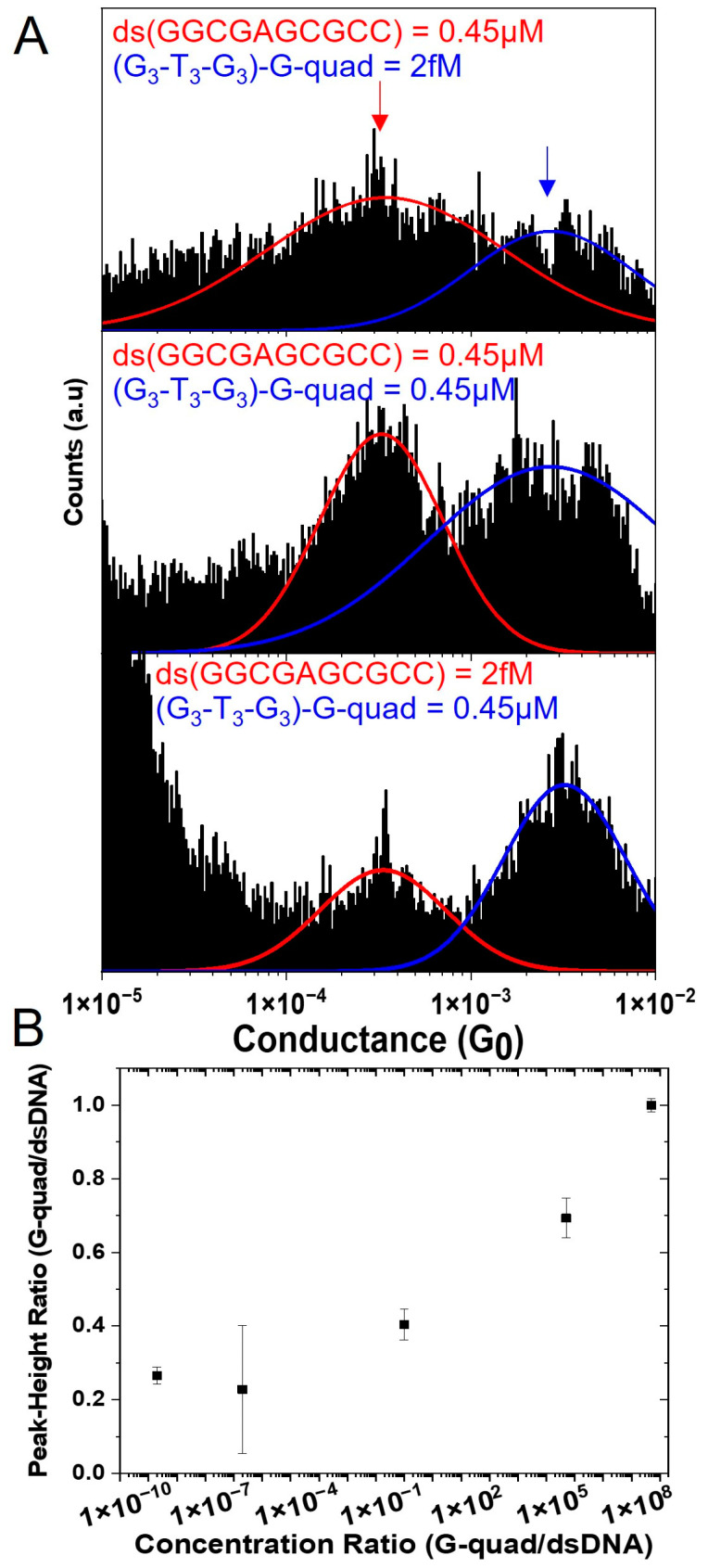
Sensitivity of the SMBJ approach to the concentration of different conformations. (**A**) Representative conductance histograms of 0.45 µM ds(GGC GAG CGCC), 2 fM (G_3_-T_3_-G_3_)-G-quad (top panel), 0.45 µM ds(GGC GAG CGCC), 0.45 µM (G3-T3-G3)-G-quad (middle panel), 2 fM ds(GGC GAG CGCC), and 0.45 µM (G3-T3-G3)-G-quad (bottom panel). (**B**) Dependence of the peak-height ratio on the concentrations ratio of the G-quad and dsDNA. Vertical error bars represent the standard error of the mean of the average peak-height ratio from two independent measurements.

Having demonstrated that the observed conductance features emerge due to the presence of multiple configurations in solution, we next considered how sensitive the conductance measurements are to the concentration of the two conformations and examined the possibility of observing low-probability configurations in complex samples. To test the sensitivity, we performed a systematic titration of the relative concentrations for the two sequences, as shown in Figure 6. First, using a constant concentration of the ds(GGC GAG CGCC) (0.45 µM), we systematically changed the concentration of the (G_3_-T_3_-G_3_)-G-quad from 2 fM to 0.45 µM (Figure 6A) and then we used a constant concentration of the (G_3_-T_3_-G_3_)-G-quad (0.45 µM) and systematically changed the concentration of ds(GGC GAG CGCC) (from 2fM to 0.45 µM). In Figure 6B, we plot the ratio of the peak amplitude (from N = 2 independent measurements) as a function of the G-quad/dsDNA concentration ratio. This indicates that not only are conductance measurements able to distinguish between the conformations but that the measurement is able to identify the presence of both conformations in a solution over an extremely large range of ratios (~16 orders of magnitude, see Supplementary File for selection percentages at each concentration). This sensitivity may allow conductance measurements to capture extremely low probability conformations within complex oligonucleotide samples.

## 4. Summary

In this work, we examined the origin of multiple conductance peaks in GC-rich DNA sequences and found that they are caused by the occurrence of both G-quadruplex and standard duplex sequences being present in the solution and that the conformation, and therefore the observed conductance, can be controlled by controlling environmental conditions. Specifically, we demonstrate that the addition of potassium chloride to the solution significantly reduces the possibility of observing anti-parallel G-quadruplex structures during conductance experiments. Moreover, we demonstrate that even though standard CD spectroscopy cannot unambiguously identify multiple conformations within a sample, SMBJ measurements are capable of detecting both G-quad and dsDNA structures in solutions over a wide range of concentration ratios, and even at concentrations down to the fM regime. These experiments suggest that single-molecule conductance measurement may offer a platform for electrical detection and the identification of various oligonucleotide conformations in complex solutions.

## Data Availability

Data available upon request from the authors.

## Supplementary Materials

The following are available online at https://www.mdpi.com/article/10.3390/biom13010129/s1, Table S1: Percentages of the selected traces in the titration experiments.
