# Supplementary material for: Mapping DNA Conformations Using Single-Molecule Conductance Measurements
Source: Biomolecules. 2023 Jan 8;13(1):129. doi: 10.3390/biom13010129 (PMC9855376; doi:10.3390/biom13010129)
Supplement: Supplementary file 1 [file biomolecules-13-00129-s001.zip › biomolecules-2097965-supplementary.pdf]

Table S1. Percentages of the selected traces in the titration experiments.

	dsDNA 2fM	dsDNA 2pM	dsDNA 0.45µM	G-quad 2fM	G-quad 2pM	G-quad 0.45µM
Trial 1	4.4%	4.44%	8.9%	5.3%	4.81%	9.27%
Trial 2	1.75%	4.05%	7.9%	5.7%	7%	9.06%

Gaussian fitting function

$$y = \frac{A\, e^{\left(\frac{-4\ln(2)(x-x_c)^2}{w^2}\right)}}{w\sqrt{\frac{\pi}{4\ln(2)}}}$$

w is the Full Width Half Maximum (FWHM)

A is the Area

x_c is the center of the peak
